# Differential expression of vitamin D associated genes in the aorta of coronary artery disease patients with and without rheumatoid arthritis

**DOI:** 10.1371/journal.pone.0202346

**Published:** 2018-08-23

**Authors:** Ingvild Oma, Ole Kristoffer Olstad, Jacqueline Kirsti Andersen, Torstein Lyberg, Øyvind Molberg, Ida Fostad, Morten Wang Fagerland, Sven Martin Almdahl, Stein Erik Rynning, Arne Yndestad, Pål Aukrust, Jon Elling Whist, Ivana Hollan

**Affiliations:** 1 Department of Pathology, Innlandet Hospital Trust, Lillehammer, Norway; 2 Institute of Clinical Medicine, University of Oslo, Oslo, Norway; 3 Department of Medical Biochemistry, Oslo University Hospital, Oslo, Norway; 4 Department of Health Sciences in Gjøvik, NTNU-Norwegian University of Science and Technology, Gjøvik, Norway; 5 Department of Rheumatology, Dermatology and Infectious Diseases, University of Oslo, Oslo, Norway; 6 Department of Oral Biology, University of Oslo, Oslo, Norway; 7 Oslo Centre for Biostatistics and Epidemiology, Research Support Services, Oslo University Hospital, Oslo, Norway; 8 Department of Cardiothoracic and Vascular Surgery, University Hospital of North Norway, Tromsø, Norway; 9 Department of cardiac surgery, Feiring Heart Clinic, Feiring, Norway; 10 Research Institute of Internal Medicine, Oslo University Hospital, Oslo, Norway; 11 Section of Clinical Immunology and Infectious Diseases, Oslo University Hospital, Oslo, Norway; 12 Department of Medical Biochemistry, Innlandet Hospital Trust, Lillehammer, Norway; 13 Department of Research, Innlandet Hospital Trust, Brumunddal, Norway; 14 Lillehammer Hospital for Rheumatic Diseases, Lillehammer, Norway; 15 Department of Medicine, Brigham and Women’s Hospital, Boston, Massachusetts United States of America; 16 Harvard Medical School, Boston, Massachusetts, United States of America; University of South Alabama Mitchell Cancer Institute, UNITED STATES

## Abstract

**Background:**

Vitamin D has an important role in the immune system, and has been linked to rheumatoid arthritis (RA) and coronary artery disease (CAD). The exact mechanisms by which vitamin D is involved in these processes are still unclear. Therefore, we wanted to search for differences in expression of genes involved in the vitamin D receptor (VDR) activation pathway and genes that are known to alter upon vitamin D stimulation, in the aortic adventitia of CAD patients with and without RA.

**Methods:**

Affymetrix microarray was used to determine gene expression profile in surgical specimens from the adventitia of the ascending aorta of CAD patients with RA (n = 8) and without RA (n = 8) from the Feiring Heart Biopsy Study.

**Results:**

We identified three vitamin D associated genes that were differentially expressed between RA and non-RA patients: Growth arrest and DNA-damage-inducible protein 45 alpha (GADD45A) (FC = 1.47; *p* = 0.006), Nuclear Receptor Co-repressor 1 (NCOR1) (FC = 1,21; *p* = 0.005) and paraoxonases 2 (PON2) (FC = -1.37; p = 0.01). High expression of GADD45A in RA tissues was confirmed by real-time qRT-PCR. GADD45A expression correlated with plasma levels of 1,25(OH)_2_D_3_ (*r*_*s*_ = 0.69; p = 0.003).

**Conclusions:**

Microarray analyses revealed higher expression of GADD45A and NCOR1; and lower expression of PON2 in the aortic adventitia of RA than non-RA patients. Further studies are needed to elucidate if and how GADD45A, NCOR1 and PON2 are involved in the development of accelerated atherosclerosis in RA. In theory, some of these factors might have proatherogenic effects whereas others might reflect an underlying vascular pathology promoting atherogenesis (such as vascular stress).

## Introduction

Rheumatoid arthritis (RA) is associated with an increased risk of cardiovascular disease (CVD) compared to the general population, mainly due to atherosclerosis [1, 2]. There are indications that both increased atheroma formation and increased plaque vulnerability might contribute to the accelerated CVD in RA [3]. Inflammation plays an important role not only in inflammatory rheumatic disease (IRD), but also in atherosclerosis (e.g, in plaque formation and plaque destabilization) [4]. Much of the research on atherosclerosis has focused on the intimal accumulation of lipids and inflammatory cells. However, there is an increasing interest in exploring the role of the adventitia and how it coordinates the immune response in atherosclerosis.

Vitamin D has been shown to have immune-modulating anti-inflammatory activities [5]. It exerts its effect through the vitamin D receptor (VDR) that has been found in most cells of the immune system, and in all the major cardiovascular cell types, including vascular smooth muscle cells (VSMCs) and endothelial cells [6, 7].

Vitamin D deficiency is common in RA patients, and it has been observed to be associated with increased CVD mortality in RA [8, 9]. Plasma levels of vitamin D have also been found to inversely correlate with RA disease activity [10]. There are indications that vitamin D might have protective effects against atherosclerosis by protecting against VSMC proliferation and migration, endothelial dysfunction, and by modulating inflammatory processes [11]. Even though vitamin D has been linked to inflammation, RA, and coronary artery disease (CAD), the underlying molecular mechanisms are still unclear.

We have previously found a higher occurrence and extent of mononuclear cell infiltrates (MCIs) and a pro-atherogenic cytokine milieu in the aortic adventitia of CAD patients with IRD compared to those without IRD. In theory, these factors might contribute to the accelerated atherosclerosis in IRD [12, 13]. The occurrence of aortic MCIs was also associated with lower levels of 1,25-dihydroxyvitamin D_3_ (1,25(OH)_2_D_3_) [14]. Surprisingly, IRD patients had higher levels of both 25-hydroxyvitamin D_3_ (25(OH)D_3_) and 1,25(OH)_2_D_3_ than non-IRD patients. The increased level of 1,25(OH)_2_D_3_ might be due to an increased conversion from 25(OH)D_3_, as indicated by a higher vitamin D ratio in IRD than non-IRD patients, whereas the increased 25(OH)D_3_ levels might be caused by the extensive vitamin D supplementation in the Norwegian IRD population [14].

VDR is a ligand dependent nuclear receptor that hetero-dimerise with its partner retinoid X receptor (RXR), then binds to vitamin D response elements (VDREs) on DNA strands and induces transcription of adjacent genes. In this study, we wanted to further investigate the potential role of vitamin D signaling in RA-related CVD by examining differences in expression of genes involved in the VDR/RXR activation pathway and genes that are known to alter upon vitamin D stimulation, in the aortic adventitia of CAD patients with and without RA.

## Methods

### Patients and biopsies

The study cohort of this cross-sectional study included 8 random patients with CAD and RA (RA group) and 8 random patients with CAD without RA (non-RA group) from the Feiring Heart Biopsy Study. From these patients, we examined specimens that were routinely removed from the ascending aorta during construction of the proximal aortacoronary veingraft anastomosis during coronary artery bypass grafting (CABG) surgery due to CAD [13]. The removed tissue contained aortic adventitia with the adjacent aortic epicardium. For patient's safety, the anastomoses were preferably made at sites with less pronounced signs of atherosclerosis [13].

All patients were Caucasians, aged >18 years, and had no history of clinically significant infection, malignancy or psoriasis. The RA patients fulfilled the American College of Rheumatology 1987 revised criteria for the classification of RA [15].

All tissue specimens were snap-frozen in liquid nitrogen and then stored at −80°C until further analyses.

### Microarray and data analyses

Total RNA was isolated from aortic specimens with TRI reagent (Sigma-Aldrich) and further purified and DNase treated using RNeasy Mini Kit (Qiagen). RNA concentration and purity was assessed with NanoDrop 1000 (Thermo Scientific). All RNA samples were stored at -80°C until analyzed.

One hundred nanograms of total RNA was subjected to a cDNA synthesis and labeling kit (GeneChip HT One-Cycle; and GeneChip HT IVT), according to the manufacturer's protocol for whole-genome gene expression analysis (Affymetrix, Santa Clara, CA, USA). Labeled and fragmented single stranded cDNAs were hybridized to GeneChip Human Genome U133A 2.0 Array (Affymetrix, Santa Clara, CA, USA) covering 18,400 transcripts and variants, including 14,500 well-characterized human genes. The arrays were washed and stained using FS-450 fluidics station (Affymetrix, Santa Clara, CA, USA). Signal intensities were detected by Hewlett Packard Gene Array Scanner 3000 (Hewlett Packard, Palo Alto, CA, USA). Microarray data are available in the Gene Expression Omnibus (www.ncbi.nlm.nih.gov/geo/) under the accession number GSE110008.

The scanned images were processed using AGCC (AffymetrixGeneChip Command Console) software. For gene expression analysis, the Affymetrix CEL files (containing probe intensities) were imported into Partek Genomics Suite software (Partek, Inc., St. Louis, MO, USA) for statistical analysis.

Robust microarray analysis (RMA) yielding normalized Log2 transformed signal intensities was applied for normalization. Gene transcripts with maximal signal values of less than 5 (Log2 values) across all arrays were removed to filter for low and non-expressed genes. Differentially expressed transcripts between groups were identified using one-way ANOVA as implemented in Partek Genomics Suite software.

Further bioinformatics analysis was conducted on the significant genes to identify functional significance by means of Ingenuity Pathways Analysis (IPA) (Ingenuity Systems, Redwood City, CA, USA). Briefly, the data set containing gene identifiers and corresponding fold changes and p-values was uploaded into the web-delivered application and each gene identifier was mapped to its corresponding gene object in the Ingenuity Pathways Knowledge Base. The data set was mined with the IPA library to extract information of the canonical VDR/RXR activation pathway, and networks were generated by using IPA graphical representations of the molecular relationships between genes and gene products.

The relative gene expression was defined as observed expression value divided by the average expression in the control group. The fold change was equal to the expression ratio when the expression ratio was ≥1. For expression ratio <1, the fold change was defined as -1/expression ratio.

To determine if there were other altered genes that could be related to the vitamin D system, in addition to those from IPA, we compared our list of significant transcripts with the list of differentially expressed genes following vitamin D stimulation from Ramagopalan *et*.*al* [16].

### Real-time qRT-PCR

To validate the results from microarray analysis, we performed real-time quantitative reverse transcription polymerase chain reactions (qRT-PCR) on the RNA from the aortic specimens, using ViiA 7 Real-Time PCR System (Applied Biosystems, Carlsbad, CA).

From the samples, 200 ng of total RNA were reverse transcribed using the Omniscript RT Kit (Qiagen Inc.). Nine μL cDNA (diluted 1:10 in H_2_O), and 1 μL of primer/probes (TaqMan Gene Expression Assays, Applied Biosystems) were added to 10 μL universal PCR master mix (TaqMan, Applied Biosystems). Each gene was run in duplicates. EEF1A1, showing a variance of 0.342 in the microarray experiments, served as endogenous control. The following assays were used: GADD45A- Hs00169255_m1 and NCOR1- Hs01094540_m1 (Applied Biosystems).

The relative amounts of mRNA for each gene were calculated by using the comparative C_T_ method [17].

### Statistical analyses

We used the Mann-Whitney U-test to examine differences in continuous distributed variables, and the Fisher mid P test to compare proportions between the two groups.

The original microarray values were Log2 transformed before further analyses. One-way ANOVA was used in the Partek Genomics Suite software to identify differentially expressed genes between CAD patients with and without RA (*p*<0.05; FC>1.10).

We used the Mann-Whitney U test to examine differences in the differentially expressed genes based on dichotomous parameters, and Spearman’s rho to estimate associations between the differentially expressed genes and selected continuous clinical and laboratory parameters from Table 1.

**Table 1:** Patients characteristics.

Characteristics	RA (n = 8)	Non-RA (n = 8)	*P* value
Age (years)	76 (13)	66 (14)	0.21
Female gender, no. (%)	3 (38)	1 (13)	0.32
Plasma 25(OH)D_3_ (nmol/L)	70 (41)	61 (32)	0.37
Plasma 25(OH)D_3_ < 50nmol/L, no. (%)	3 (38)	3 (38)	0.61
**Plasma 1,25(OH)**_**2**_**D**_**3**_ **(pmol/L)**	**147 (92)**	**85 (30)**	**0.007**
MCIs in aortic adventitia, no. (%)	1 (14)	2 (25)	0.57
CRP (mg/L)	10 (15.9)	4.3 (4.2)	0.06
ESR (mm/h)	23 (32)	13 (17)	0.10
**Pentraxin 3 (ng/ml)**	**2.1 (1.4)**	**1.2 (1.4)**	**0.02**
BMI (kg/m2)	27 (6.0)	27 (3.8)	0.60
Diabetes, no. (%)	0 (0)	1 (13)	0.500
Hypertension, no. (%)	4 (50)	5 (63)	0.66
Duration of CAD (months)	48 (171)	60 (168)	0.75
Current smoker, no. (%)	1 (13)	1 (13)	0.47
Previous smoker, no. (%)	3 (38)	4 (50)	0.66
Daily vitamin and mineral suppl., no. (%)	3 (60)	1 (14)	0.08
Daily fishoil suppl., no. (%)	4 (80)	4 (57)	0.22
Hyperlipidemia, no. (%)	7 (88)	8 (100)	0.500
LDL (mmol/L)	2.9 (1.1)	2.8 (0.5)	0.37
HDL (mmol/L)	1.2 (0.7)	1.2 (0.2)	0.83
Total cholesterol (mmol/L)	4.7 (1.8)	4.5 (0.9)	0.77
Triglycerides (mmol/L)	2.0 (1.6)	1.7 (0.3)	0.62
**Creatinine (**μ**mol/L)**	**84 (20)**	**94 (24)**	**0.03**
eGFR (mL/min/1.72 m^3^)	77 (20)	68 (20)	0.07
Acetylsalycyclic acid, no. (%)	8 (100)	7 (88)	0.500
**Glucocorticosteroids, no. (%)**	**4 (50)**	**0 (0)**	**0.04**
Statins, no. (%)	7 (88)	8 (100)	0.500
ACE inhibitors, no. (%)	2 (25)	3 (38)	0.64
**DMARDs, no. (%)**	**7 (88)**	**0 (0)**	**<0.001**
NSAIDs, no. (%)	1 (13)	0 (0)	0.500
Coxibs, no. (%)	2 (25)	0 (0)	0.23
Betablockers, no. (%)	8 (100)	6 (75)	0.23
Duration of RA (years)	20 (29)	NA	NA
PtGA* (cm)	0.2 (3.3)	NA	NA
PGA* (cm)	0.5 (1.4)	NA	NA

Unless indicated otherwise, values are given as median (interquartile range).

Abbreviations: RA, rheumatoid arthritis; MCI, mononuclear cell infiltrate; CRP, C-reactive protein; ESR, erythrocyte sedimentation rate; BMI, body mass index; CAD, coronary artery disease; suppl., supplementation; LDL, low density lipoprotein; HDL, high density lipoprotein; eGFR, estimated glomerular filtration rate; ACE, angiotensin-converting enzyme; DMARD, disease-modifying anti-rheumatic drug; NSAID, non-steroidal anti-rheumatic drug; coxib, cyclooxygenase-2 selective inhibitor; PtGA, patient’s global assessment score of disease activity; PGA, physician`s global assessment score of disease activity; NA, Not applicable.

*On a 10-cm visual analogue scale.

Statistical comparison of real-time qRT-PCR data was performed with the unpaired Student’s t-test.

Statistical analyses were performed using SPSS, version 23 (SPSS, Chicago, IL, USA), Microsoft Excel 2011 (Microsoft Corporation, Redmond, WA, USA) and Partek Genomics Suite software (Partek, Inc., St. Louis, MO, USA).

The level of statistical significance was set at 0.05, and all statistical tests were 2-sided.

### Ethics approval and consent to participate

The Regional Committee for Medical and Research Ethics in Norway approved the study protocol, reference ID S-00243. All patients gave their written informed consent.

## Results

### Patients characteristics

The characteristics for the study cohort, including medication are shown in Table 1. The distribution of traditional cardiovascular risk factors was similar between the RA and non-RA group, but the RA patients had higher levels of the inflammatory parameter pentraxin 3 (PTX3). The level of 25(OH)D_3_ and the frequency of vitamin D deficiency (25(OH)D_3_ <50 nmol/L) was similar in both groups, but the RA group had higher 1,25(OH)_2_D_3_ levels than the non-RA group. (Table 1). The occurrence of MCIs in their aortic adventitia was not statistically different.

### Differential expression of vitamin D associated genes in the aortic adventitia

**Fig 1:**
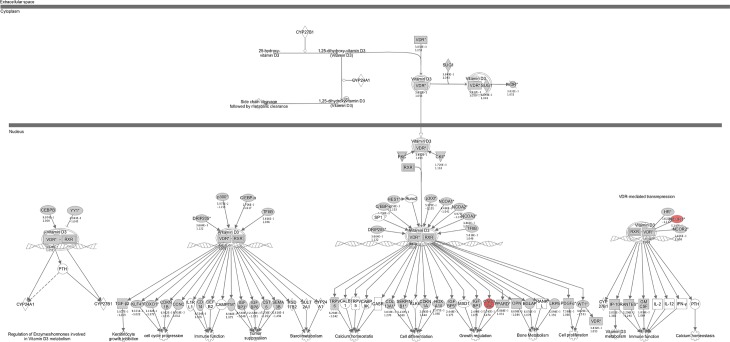
Graphical representation of the VDR/RXR activation pathway from Ingenuity Pathway Analysis (IPA). The data set containing gene identifiers and corresponding fold changes and p-values generated from one-way ANOVA analysis in Partek Genomic Suite Software was uploaded into the web-delivered IPA application. Each gene identifier was mapped to its corresponding gene object in the Ingenuity Pathway Knowledge Base. The data set was mined with the IPA library to extract information of the canonical VDR/RXR activation pathway, and networks were generated by using IPA graphical representations of the molecular relationships between genes and gene products. Red color represent genes that are upregulated; grey: genes from the dataset that did not pass the analysis cutoffs; white: genes from the Ingenuity Pathway Knowledge Base that are not part of the dataset.

**Fig 2:**
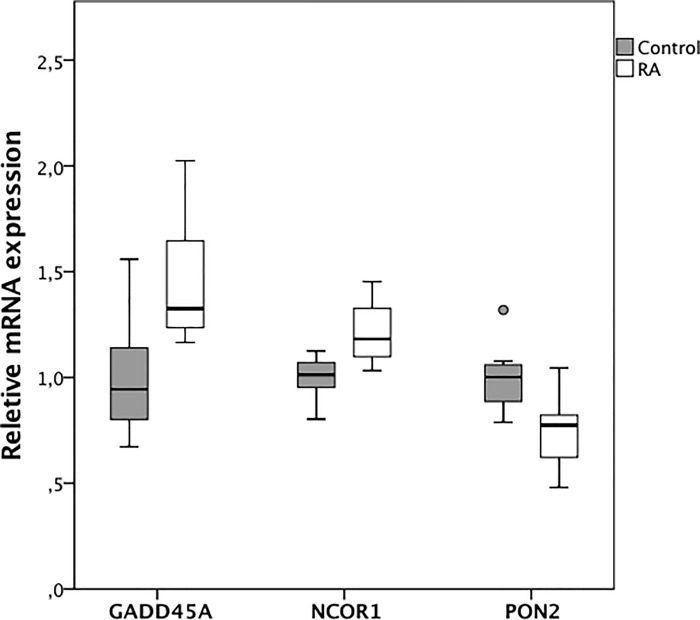
Box-and-whiskers plot of the relative mRNA expression of GADD45A, NCOR1 and PON2 from microarray analysis. Solid horizontal lines represent median values, boxes interquartile range (IQR), and whiskers minimum and maximum values (excluding outliers); °outliers (1.5–3 × IQR).

Using Affymetrix microarray, a total of 15586 transcripts were identified. When filtered for low and non-expressed genes, the number of transcripts were reduced to 10 891 transcripts, of which 201 were differentially expressed between the two groups. IPA determined two genes within the VDR/RXR activation pathway: Growth arrest and DNA-damage-inducible protein 45 alpha (GADD45A) (FC = 1.47; *p* = 0.006) and Nuclear Receptor Co-repressor 1 (NCOR1) (FC = 1.21; *p* = 0.005), that where both up-regulated in RA patients (Figs 1 and 2).

Expression of VDR and its partner RXR were not altered between the two groups. Transcripts of cytochrome P450 27B1 (CYP27B1) and cytochrome P450 24A1 (CYP24A1), that catalyzes the hydroxylation of 25(OH)D_3_ to 1,25(OH)_2_D_3_ and inactivates 1,25(OH)_2_D_3_, respectively, were not identified in this study.

When we compared our list of 201 differentially expressed transcripts with the list of genes that have been previously reported to be altered upon vitamin D stimulation by Ramagopalan *et*.*al* [16], the only gene that was present on both lists was paraoxonases 2 (PON2), and the expression of PON2 was lower in RA patients than in non-RA patients (FC = -1.37; p = 0.014) (Fig 2).

**Fig 3:**
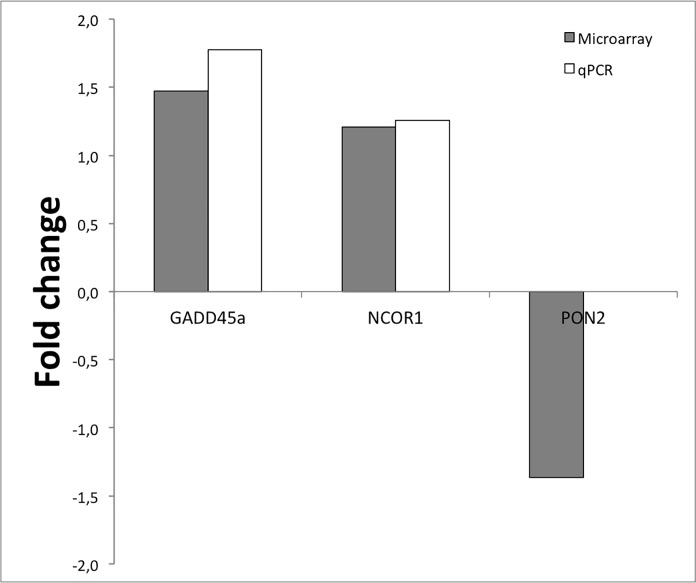
Fold change of GADD45A, NCOR1 and PON2 from microarray and real-time qRT-PCR.

Due to limiting amounts of RNA from the individual aortic biopsies it was not possible to perform extensive real-time qRT-PCR validations. We were, however, able to confirm higher expression of GADD45A in RA tissues (FC = 1.77; p = 0.007). There was also a trend towards higher NCOR1, but this did not reach statistical significance (FC = 1.26; p = 0.172) (Fig 3).

### Correlations between differentially expressed genes and clinical and laboratory variables

We searched for correlations between GADD45A, NCOR1 and PON2 and clinical and laboratory parameters listed in Table 1.

#### GADD45A and the correlation to other factors

There was a strong positive correlation between the expression of GADD45A and plasma 1,25(OH)_2_D_3_ (*r*_*s*_ = 0.69, 95% CI 0.29 to 0.88; p = 0.003), moderate positive correlation with eGFR (*r*_*s*_ = 0.50, 95% CI 0.006 to 0.80; p = 0.048) and age (*r*_*s*_ = 0.53, 95% CI 0.04 to 0.81; p = 0.036), and a moderate negative correlation between GADD45A and serum creatinine (*r*_*s*_ = -0.58, 95% CI -0.84 to -0.12; p = 0.019).

GADD45A expression was higher in patients currently using DMARDs (FC = 1.25; p = 0.039) and NSAIDs or coxibs (FC = 1.52; p = 0.013).

#### NCOR1 and the correlation to other factors

NCOR1 expression was also higher in patients currently using DMARDs (FC = 1.20; p = 0.002). In addition, there was a moderate positive correlation between NCOR1 expression and PTX3 (*r*_*s*_ = 0.52, 95% CI 0.04 to 0.81; p = 0.037).

#### PON2 and the correlation to other factors

The expression of PON2 was negatively related to NCOR1 expression (*r*_*s*_ = -0.52, 95% CI -0.81 to -0.04; p = 0.037). Furthermore, there was a tendency towards a negative association between PON2 and 1,25(OH)_2_D_3_ (*r*_*s*_ = -0.43, 95% CI -0.76 to 0.08, p = 0.094) and the current use of NSAIDs or coxibs (FC = -1.28; p = 0.039).

## Discussion

From the microarray results in this study, we revealed a differential expression of three vitamin D associated genes in the aortic adventitia of CAD patients with and without RA: while the expression of GADD45A and NCOR1 was higher, the expression of PON2 was lower in RA than non-RA patients.

### GADD45A

GADD45A is a regulatory molecule which aim is primarily to protect cells and ensure survival by inducing cell cycle arrest and DNA repair [18]. GADD45A expression can be upregulated by environmental stresses such as hypoxic, osmotic and heat stress, ionizing and UV-radiation and chemical mutagens [18, 19]. GADD45A also has an adjacent VDRE and therefore 1,25(OH)_2_D_3_ can upregulate the expression of GADD45A [20].

GADD45 proteins have been linked to autoimmunity. An increase in GADD45A expression has been observed in T-cells from systemic lupus erythematous (SLE) patients [21], and GADD45A^-/-^ mice spontaneously develop an autoimmune disease similar to human SLE [22].

The increased GADD45A expression in the aortic wall of RA patients could potentially indicate a pathogenic role for GADD45. However, an up-regulation of GADD45A could also represent a protective and counteracting mechanism, and one can hypothesize that increase in GADD45A expression could be caused by enhanced oxidative, inflammatory and metabolic stress in the aortic wall of RA patients, which counteract the induction of DNA damage, apoptosis and cell death. In theory, the enhanced vascular stress might be secondary to the chronic autoimmune process, to factors that play a pathogenic role in RA, or to RA treatment. Although one study showed no changes in GADD45A expression in peripheral blood cell subpopulations after one month of methotrexate treatment in patients with early RA [23], there are indications that some drugs, including NSAIDs, can upregulate GADD45A [18, 19]. In our study, the expression of GADD45A was higher in patients using DMARDs, and NSAIDs or coxibs. Unfortunately, due to our low sample size, we were not able to explore if the difference in GADD45A expression between RA and non-RA patients was independent of the used medications, and vice versa.

Based on these results, one might speculate that increased GADD45A expression might play a role in the accelerated atherosclerosis of patients with RA. Thus, further studies are necessary to elucidate the role of GADD45A in atherosclerosis and premature atherosclerosis in RA.

### NCOR1

NCOR1 is a co-repressor that contains nuclear receptor interacting domains and interacts with various nuclear receptors, including VDR. NCOR1 recruits histone deacetylase enzymes (HDACs) which deacetylase histone-tails and thereby condense the DNA structure and prevent gene-transcription [24]. NCOR1 has an important role as a gene-specific integrator of positive and negative signals that controls inflammation [24, 25].

1,25(OH)_2_D_3_ has been shown to increase NCOR1 gene expression [20], as well as to increase the number of binding sites for NCOR1 on VDRs [26].

The microarray analyses in our study indicated that RA patients had higher levels of NCOR1 in the aortic adventitia than non-RA patients. As NCOR1 has an important role as a gene-specific integrator of positive and negative signals that controls inflammation, the up-regulation of NCOR1 could potentially have both anti-inflammatory and pro-inflammatory effects.

However, this finding was not confirmed by real-time qRT-PCR. We believe that this may be explained by differential primer specificities of the microarray and the PCR. NCOR1 occurs in several isoforms. In the microarray analysis, four different NCOR1 transcripts we detected; and only one of these were differentially expressed between the RA and non-RA groups. The primer used for real-time qRT-PCR did not exclusively bind to this specific transcript, but detected other isoforms as well. Unfortunately, due to limiting amounts of cDNA, we were not able to perform further PCR analyses to test this assumption. Therefore, further studies on this topic are warranted.

### PON2

PON2 is a ubiquitously expressed intracellular protein that exhibits antioxidant properties. The antioxidant effect of PON2 is believed to play a major role in preventing the atherosclerotic process [27]. Indeed, PON2-deficient mice fed with a high-cholesterol diet have been observed to develop significantly larger atherosclerotic lesions compared to their wild-type counterparts. Even though serum levels of high-density lipoprotein (HDL), triglycerides and glucose were equal, serum levels of very low-density lipoprotein (VLDL) and low-density lipoprotein (LDL) were significantly lower in PON2-deficient mice. In addition, their LDL exhibited enhanced pro-inflammatory properties, and the mice had signs of increased oxidative stress along with an exacerbated inflammatory response from PON2-deficient macrophages [28]. HeLa cells overexpressing PON2 show less intracellular oxidative stress when exposed to H_2_O_2_ or oxidized phospholipids and are less effective in oxidizing and modifying LDL [29]. Genetic polymorphisms of PON2 have also been found to be associated with CVD [30].

Thus, the reduced vascular expression of PON2 might potentially contribute to the accelerated atherogenesis in RA, e.g., via increased oxidative stress in vessel walls and increased oxidation of LDL leading to lipid accumulation and plaque development.

There is a lack of knowledge of PON2 in general, and further studies on how PON2 is involved in atherosclerosis, how it is associated with vitamin D and how it affects RA patients are warranted.

### Limitations

Our study has several limitations. First, owing to the cross-sectional design of our study, inferences on the direction of causality cannot be drawn.

Second, owing to a relatively small sample size, we were not able to perform multiple regression analysis, and both type I and type II errors might have occurred. For example, we do not know if the correlation between PTX3 and GADD45A was due to their direct relationship or due to the effect of RA as PTX3 was higher in RA than non-RA patients. The same applies for use of anti-inflammatory drugs. However, it is important to point out that access to surgical adventitial specimens from RA and non-RA patients is extremely limited. Furthermore, due to post-mortem processes, surgical specimens are superior to autopsy specimens. Thus, in spite of the low sample size, the study has an important hypothesis generating potential. Being the first study to examine the expression of vitamin D related genes in the aortic adventitia in RA and non-RA patients with CAD, this study may give directions for further research.

Third, due to small amounts of mRNA, we were not able to confirm microarray results regarding PON2, and to investigate if the disagreement between microarray and real-time qRT-PCR concerning NCOR-1 could be explained by the expression of different isoforms of NCOR1.

## Conclusions

The microarray analysis revealed differential expression of three vitamin D associated genes in the aortic adventitia in RA and non-RA patients with CAD: while the expression of GADD45A and NCOR1 was higher, the expression of PON2 was lower in RA patients. However, due to limited amount of mRNA, only the differential expression of GADD45A was confirmed by real-time qRT-PCR.

However, although it is possible that the up-regulation of GADD45A and NCOR1 is protective, and the down-regulation of PON2 is harmful in relation to vascular tissue injury, these issues are far from clear and needs to be further studied in forthcoming experimental and clinical studies.
